# CMBD: a manually curated cancer metabolic biomarker knowledge database

**DOI:** 10.1093/database/baaa094

**Published:** 2021-03-09

**Authors:** Jing Chen, Xingyun Liu, Li Shen, Yuxin Lin, Bairong Shen

**Affiliations:** Institutes for Systems Genetics, Frontiers Science Center for Disease-related Molecular Network, West China Hospital, Sichuan University, Chengdu, Sichuan 610041, China; The School of Science, Kangda College of Nanjing Medical University, Lianyungang, Jiangsu 222000, China; Institutes for Systems Genetics, Frontiers Science Center for Disease-related Molecular Network, West China Hospital, Sichuan University, Chengdu, Sichuan 610041, China; Institutes for Systems Genetics, Frontiers Science Center for Disease-related Molecular Network, West China Hospital, Sichuan University, Chengdu, Sichuan 610041, China; Department of Urology, The First Affiliated Hospital of Soochow University, Suzhou, Jiangsu 215006, China; Institutes for Systems Genetics, Frontiers Science Center for Disease-related Molecular Network, West China Hospital, Sichuan University, Chengdu, Sichuan 610041, China

## Abstract

The pathogenesis of cancer is influenced by interactions among genes, proteins, metabolites and other small molecules. Understanding cancer progression at the metabolic level is propitious to the visual decoding of changes in living organisms. To date, a large number of metabolic biomarkers in cancer have been measured and reported, which provide an alternative method for cancer precision diagnosis, treatment and prognosis. To systematically understand the heterogeneity of cancers, we developed the database CMBD to integrate the cancer metabolic biomarkers scattered over literatures in PubMed. At present, CMBD contains 438 manually curated relationships between 282 biomarkers and 76 cancer subtypes of 18 tissues reported in 248 literatures. Users can access the comprehensive metabolic biomarker information about cancers, references, clinical samples and their relationships from our online database. As case studies, pathway analysis was performed on the metabolic biomarkers of breast and prostate cancers, respectively. ‘Phenylalanine, tyrosine and tryptophan biosynthesis’, ‘phenylalanine metabolism’ and ‘primary bile acid biosynthesis’ were identified as playing key roles in breast cancer. ‘Glyoxylate and dicarboxylate metabolism’, ‘citrate cycle (TCA cycle)’, and ‘alanine, aspartate and glutamate metabolism’ have important functions in prostate cancer. These findings provide us with an understanding of the metabolic pathway of cancer initiation and progression.

**Database URL**: http://www.sysbio.org.cn/CMBD/

## Introduction

Cancer is a complex disease, associated with genetic, proteinic, metabolic and epigenetic changes and their interactions during its initiation and progression (1–4). Aerobic glycolysis or the Warburg effect in some cancer cells is the beginning of research in cancer metabolism and the basis for the positron emission tomography scan to observe metabolic processes in modern medicine (4–6). In addition to deregulating cellular energetics as one of the 10 hallmarks of cancer, subsequent metabolic changes are of interest to researchers (7). Such as, advances in understanding of polyamines metabolism leads to a novel anticancer targeting strategy (8). The interactions between oncogenic signaling, lipid metabolism and epigenetics indicate highly flexible and robust cancer cybernetic networks (9). Metabolic enzymes can be associated with highly invasive and metastatic cancer. The activity of lactate dehydrogenase and catalase could help define aggressive breast cancers (10). Glucose-6-phosphate dehydrogenase upregulated by androgen receptor signaling plays a vital role in prostate cancer cell growth and maintenance (11). Nowadays, cancer metabolism, including abnormal lipid, amino acid, nucleotide, nutrient and non-nutrient metabolism can provide a new strategy for cancer detection, treatment and management.

Biomarkers refer to the measurable and evaluable indicators of normal biological process, pathogenic process, or pharmacologic responses to a therapeutic intervention (12, 13). Biomarkers could be classified as molecular, physiological or image biomarkers, etc. (14). Cancer metabolic biomarkers in the present work are defined as genes/proteins regulating or participating in metabolic processes, intermediates or end products (usually as metabolites) in the metabolic pathways as well as some other small chemical molecules detected in tissues and body fluids, which could indicate the biological or disease states. For example, BEC index (β-F1/Hsp60/GAPDH), proteomic markers of the metabolic phenotype, may monitor the prognosis of breast cancer patients (15). High spermine concentration in prostatectomy specimens is associated with longer recurrence-free survival (16, 17). Weight, a typical physiological biomarker, is a potential independent predictor of relapse and death in acute myeloid leukemia (AML) patients. Therefore, addressing pretransplant nutritional interventions may reduce AML relapse rates (18). Image biomarkers are mainly the parameters in the visual images obtained through imaging techniques. They can quantify tumor metabolic process for further analysis (5, 19). Standardized uptake values (SUV), metabolic tumor volume (MTV), total lesion glycolysis (TLG) are sensitive prognosis biomarkers for cancer documented in the literature (20–22). By the development of modern technologies, a large number of metabolic biomarkers in different tumors are measured and reported with potential applications in cancer diagnosis, treatment and prognosis (23, 24).

However, to our knowledge, there is no resource integrating the existing metabolic biomarkers for human cancers. To provide a systems biological view on cancer metabolism biomarkers, we have developed the first database that integrates these biomarkers scattered over published literatures in PubMed. It contains 438 manually curated relationships between 282 biomarkers and 76 cancer subtypes among 18 tissues reported in 248 literatures. Comprehensive information about cancers, biomarkers, reference, samples and their relationships is available at http://www.sysbio.org.cn/CMBD/. This resource will be a useful tool for researchers and clinicians to query and retrieve metabolic biomarker information associated with different cancers.

## Methods

### The overview of CMBD construction and analysis

**Figure 1.**
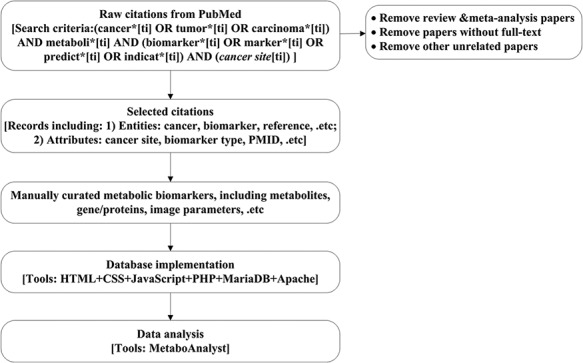
Work flow chart. Here, the cancer site will be specific to cancer site searched, e.g. breast, prostate and lung. Details for search criteria and result are shown in supplementary file 1.

The CMBD construction and analysis includes the following steps as shown in Figure 1.

1. Search and collect the cancer metabolism biomarkers associated citations from PubMed;
2. Screen and standardize the annotations for the metabolism biomarkers;
3. Manually curate biomarkers according to database design;
4. Implement the CMBD;
5. Analyze the data.

### Data collection

We searched literatures published before December 31, 2019 in English with keywords (‘cancer’, ‘metabolism’, and ‘biomarker’) and their synonyms in the PubMed database, removing reviews, meta-analysis papers, papers without full text and other unrelated papers. Details for search criteria and results are shown in online supplementary material file 1.

### Data standardization and annotation

We selected literatures describing metabolic biomarkers for human cancers. Then, we chose the necessary information to record a biomarker–cancer–reference relationship in detail. Data standardization and annotation are essential for sharing data. For cancers, not only the disease’s specific names and tissues/organs are listed in the original literature, but also their International Classification of Disease (ICD, 10th revision) codes are included for further analysis. For single biomarkers, their chemical names or abbreviations in CMBD are extracted from Gene, UniProt, HMDB, Wiki and PubMed; unique entry IDs are given in CMBD to avoid duplicate records because of synonyms. For combined biomarkers, their names are commonly abbreviated to expressions or ‘model_PMID_number_components’; components of combined biomarkers are recorded and stored separately for further analysis. Table 1 shows the examples for the biomarker annotation. The details on the standardization of biomarker names are available at the database webpage http://www.sysbio.org.cn/CMBD/.

**Table 1.** Examples of biomarkers standardization and annotation

Names in literatures	Name in database	Type	Consult databases	Entry ID
12-keto-LTB4	12-keto-LTB4	fatty acyls	HMDB	HMDB0004234
c-MYC	c-MYC	gene	Gene	4609
c-Met	c-Met	protein	UniProt	P08581
N-telopeptide/N-terminal telopeptide/NTx	NTx	not classified	WIKI	N-terminal telopeptide
The area under the curve (AUC) of cumulative SUV histograms (CSH)	AUC-CSH	not classified	PubMed	26 063 655
Expression of TS × DPD	TS × DPD	not classified	Custom table	122 123
A panel of four metabolites (kynurenine, acetylcarnitine, PC(42:11), and LPE (22:0/0:0))	model_28389631_4_components	not classified	Custom table	114 115 116 117
Lactate/lactic acid	Lactate	Hydroxy acids and derivatives	HMDB	HMDB0000190

### Database design

**Figure 2.**
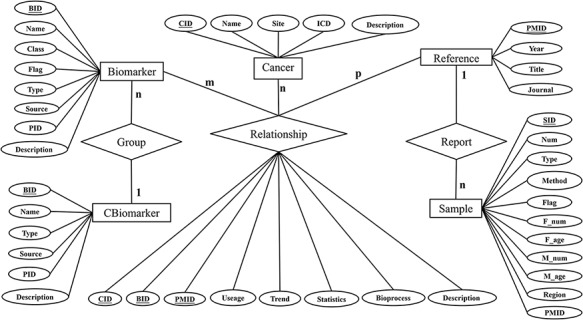
Entity-Relation diagram.

The database was designed based on the entity relationship model abstracted from the requirements information. The model consists of six entities (‘Cancer’, ‘Biomarker’, ‘Cbiomarker’ (combined biomarker), ‘Reference’, ‘Relationship’, and ‘Sample’). Their specific attributes and relationships are visualized in the Entity-Relation diagram as shown Figure 2. We manually curated the collected data according to the entries.

### Database implementation and data analysis

Several tools were utilized here for database construction. The main development tool is XAMPP v3.2.2, which includes Apache 2.4.23 as the web server, MariaDB 10.1.19 as the database management system and PHP as the server-side scripting language to execute dynamic SQL queries. HTML, CSS and JavaScript were selected as the client-side languages for front-end design. MetaboAnalyst (http://www.metaboanalyst.ca/) (23 ) was used for comprehensive metabolic data analysis.

## Results

### The utility of the database

**Figure 3.**
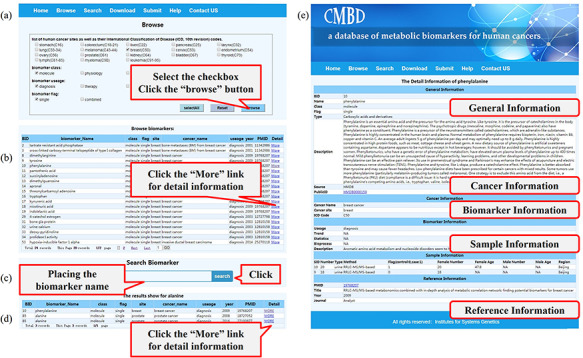
A screenshot of the utility of CMBD. (a) The browse page, biomarkers are classified by their site, class, usage and flag reported in the literature; (b) the list of items matching the filter criteria; (c) search page; (d) the list of items matching fuzzy retrieval; (e) detail information about biomarkers.

CMBD provides a friendly web interface for users to access the database (Figure 3). On the ‘browse’ page, users can click the checkbox to specify filters (tissue site, class, usage and flag) as needed and then click the ‘Browse’ button (Figure 3a); the items matching the filter criteria will be listed on a new page and users can get a biomarker in detail by clicking the ‘More’ link (Figure 3b). The page will display the information about the cancer, biomarker, sample and reference, etc. On the ‘search’ page, users can query the database by biomarker name (Figure 3c). Results for fuzzy retrieval are also listed on a new page and users can get the information by clicking the ‘More’ link (Figure 3d). By clicking the links on the biomarker information webpage, the information for the biomarker can be displayed from the corresponding public databases (Figure 3e). Users can download or submit data on the ‘Download’ or ‘Submit’ page, respectively. More details about the utility of CMBD are shown on the online ‘Help’ page.

### Classification and statistics of biomarkers

**Figure 4.**
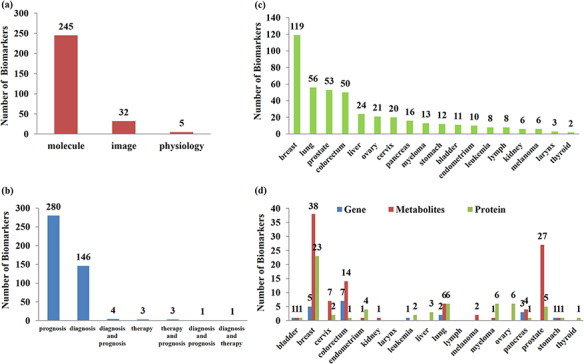
Classification and statistics of biomarkers. (a) Type of biomarkers; (b) usage distribution of biomarkers; (c) site distribution of biomarkers; (d) classification distribution of single molecular biomarkers in different sites.

CMBD documents 438 manually curated relationships between 282 biomarkers and 76 cancer subtypes among 18 tissue sites reported in 248 literatures. Figure 4a shows the type distribution of 282 biomarkers and most of them are molecular biomarkers. Figure 4b shows the usage distribution of biomarkers in 438 relationships and more than half of them are for prognosis assessment. Figure 4c shows the tissue sites distribution of biomarkers in 438 relationships. Figure 4d shows the classification distribution of 185 single molecular markers in 438 relationships among different tissue sites, and most of the single molecular biomarkers are metabolites.

**Figure 5.**
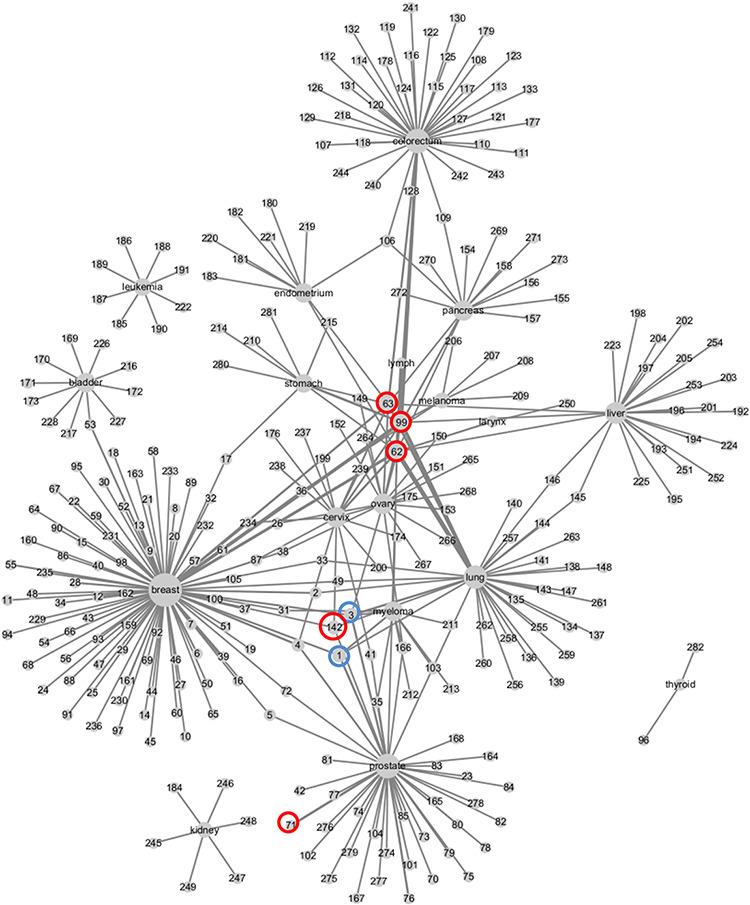
Weighed network of cancers and metabolic biomarkers. Nodes represent cancers and biomarkers, labeled by cancer site and biomarkers’ ID in CMBD. Cancer node and biomarker node are linked by an edge if they are reported in a literature. The size of cancer node represents the number of biomarker nodes linked to it, and the width of edge represents the frequencies that the cancer and the biomarker are reported in literatures.

Based on the CMBD, we constructed the weighed network for cancer types and metabolic biomarkers as shown in Figure 5. Breast cancer has the most reported biomarkers. Image parameters including SUV (99), MTV (62), and TLG (63) are reported for many cancers. Bone metabolic markers including cross-linked carboxy-terminal telopeptide of type I collagen (3), bone alkaline phosphatase (1), are also reported in many cancers, which are of great value for the detection of bone metastasis from cancer. Glutamate (142) is reported in three cancers and spermine (71) is reported in prostate cancer four times.

### Metabolic pathway analysis for cancer biomarkers

Breast cancer and prostate cancer are prevalent in women and men, respectively. Both are hormone dependent and cancers of the reproductive system. Considering this and the current data in CMBD, we chose metabolites biomarkers for breast cancer and prostate cancer, and then performed pathway analysis on the MetaboAnalyst website (http://www.metaboanalyst.ca) using their HMDB ID (online supplementary file 2) individually, keeping all the other settings on the default parameters. Pathway results of metabolites for breast cancer and prostate cancer are shown in Tables 2 and 3. The pathway impact is calculated as the cumulative percentage from hits normalized importance (25).

**Table 2.** Pathway results of metabolites for breast cancer (*P *< 0.05)

No.	Pathway name	Total	Hits	Raw p	Impact
1	Primary bile acid biosynthesis	46	5	0.00091398	0.0902
2	Phenylalanine, tyrosine and tryptophan biosynthesis	4	2	0.0017167	1
3	Aminoacyl-tRNA biosynthesis	48	4	0.0084164	0
4	Phenylalanine metabolism	10	2	0.012069	0.35714
5	Neomycin, kanamycin and gentamicin biosynthesis	2	1	0.034546	0

**Table 3.** Pathway results of metabolites for prostate cancer (*P *< 0.05)

No.	Pathway name	Total	Hits	Raw p	Impact
1	Aminoacyl-tRNA biosynthesis	48	7	6.65E-07	0
2	Valine, leucine and isoleucine biosynthesis	8	3	8.43E-05	0
3	Alanine, aspartate and glutamate metabolism	28	4	0.00027464	0.11378
4	Glyoxylate and dicarboxylate metabolism	32	3	0.0061909	0.13757
5	Valine, leucine and isoleucine degradation	40	3	0.011588	0
6	Citrate cycle (TCA cycle)	20	2	0.023723	0.12311
7	Glutathione metabolism	28	2	0.044529	0.08873

Aminoacyl-tRNA biosynthesis is identified as a significant pathway in both breast and prostate cancers. Aminoacyl-tRNAs are the essential substrates for translation and are related to genetic information processing (26). Aminoacyl-tRNA synthetases mutations also occur in many human diseases but the biological mechanism is still unknown (27).

‘Phenylalanine, tyrosine and tryptophan biosynthesis’, ‘phenylalanine metabolism’ and ‘primary bile acid biosynthesis’ are screened as significant pathways with high impact in breast cancer. Tryptophan metabolism is influenced by estrogens and it is abnormal in patients with breast cancer (28, 29). Furthermore, tryptophan oxidation via the kynurenine pathway plays an important role in allowing tumor cells to escape immune surveillance in the tumor-bearing host and in creating an immunosuppressive environment (29–31). Bile acids regulate lipids, carbohydrates, and their metabolism. Previous studies indicate that bile acids and their derivatives mediate apoptosis via a p53-independent pathway (32, 33). Bile acid biosynthesis has been reported as a new target for the treatment of breast cancer (34, 35).

‘Glyoxylate and dicarboxylate metabolism’, ‘citrate cycle (TCA cycle)’, and ‘alanine, aspartate and glutamate metabolism’ are identified as significant pathways with high impact in prostate cancer. The citrate cycle, regulated by testosterone and prolactin, can influence alanine, aspartate and glutamate metabolism, as it provides precursors for anabolism of these amino acids, α-ketoglutarate for alanine, oxaloacetate for aspartate, and oxaloacetate for alanine by converting to pyruvate first (36, 37). Compared with normal cells, prostate cancer cells are more sensitive to citric acid, an intermediate product of the citric acid cycle, and the intracellular citric acid content is lower, probably because elevated testosterone promotes citric acid metabolism. The significant decrease in citric acid level in the prostate promoted the use of magnetic resonance spectroscopy for early biopsy of prostate cancer, and provided important clues for the use of new non-invasive early screening methods (36).

## Discussion and conclusion

Based on our knowledge, CMBD is the first manually curated database containing comprehensive metabolic biomarker information for human cancers. It shows good evidence for clinicians to improve diagnosis, treatment and prognosis of cancer patients. For example, lower levels of lactate were observed in breast cancer patients with long-term survival (38–40), and high spermine and citrate concentrations were associated with longer recurrence-free survival for prostate cancer patients (16, 41). However, there is not yet enough data to compare the metabolic differences and similarities in various cancers. We just compared the metabolic pathway differences in breast cancer and prostate cancer. The mechanisms behind these differences are as yet unknown. This could be improved if more metabolism biomarkers are reported and accumulated. Considered to be an interactive website, CMBD will be updated regularly according to newly available biomarkers and user-submitted data. A systems biological perspective on the heterogeneity of cancer metabolism could be expected.

In conclusion, CMBD will be a useful tool for researchers and clinicians to query and retrieve metabolic alterations in tumors and to manage personalized cancer treatment.

## Supplementary Material

baaa094_Supp
