# Supplementary material for: CMBD: a manually curated cancer metabolic biomarker knowledge database
Source: Database (Oxford). 2021 Mar 9;2021:baaa094. doi: 10.1093/database/baaa094 (PMC7947571; doi:10.1093/database/baaa094)
Supplement: baaa094_Supp [file baaa094_supp.zip › Supplemental file 1.doc]

**Table S1: Details for literature search and inclusion**

| No | site | search criteria | search result | included |
| --- | --- | --- | --- | --- |
| 1 | bladder | bladder[ti] AND (cancer*[ti] OR tumor*[ti] OR carcinoma*[ti]) AND metaboli*[ti] AND (biomarker*[ti] OR marker*[ti] OR predict*[ti] OR indicat*[ti])AND ("1900/01/01"[DP] : "2019/12/31"[DP]) | 12 | 8 |
| 2 | brain | brain[ti] AND (cancer*[ti] OR tumor*[ti] OR carcinoma*[ti]) AND metaboli*[ti] AND (biomarker*[ti] OR marker*[ti] OR predict*[ti] OR indicat*[ti])AND ("1900/01/01"[DP] : "2019/12/31"[DP]) | 6 | 0 |
| 3 | breast | breast[ti] AND (cancer*[ti] OR tumor*[ti] OR carcinoma*[ti]) AND metaboli*[ti] AND (biomarker*[ti] OR marker*[ti] OR predict*[ti] OR indicat*[ti])AND ("1900/01/01"[DP] : "2019/12/31"[DP]) | 107 | 51 |
| 4 | cervix | (cervical[ti] OR cervix[ti])AND(cancer*[ti] OR tumor*[ti] OR carcinoma*[ti]) AND metaboli*[ti] AND (biomarker*[ti] OR marker*[ti] OR predict*[ti] OR indicat*[ti])AND ("1900/01/01"[DP] : "2019/12/31"[DP]) | 16 | 11 |
| 5 | colorectum | (colorectal[ti] OR colon[ti] OR rectal[ti] OR colorectum[ti] OR bowel[ti] )AND (cancer*[ti] OR tumor*[ti] OR carcinoma*[ti]) AND metaboli*[ti] AND (biomarker*[ti] OR marker*[ti] OR predict*[ti] OR indicat*[ti])AND ("1900/01/01"[DP] : "2019/12/31"[DP]) | 67 | 28 |
| 6 | endometrium | (endometrial[ti] OR endometrium[ti])AND (cancer*[ti] OR tumor*[ti] OR carcinoma*[ti]) AND metaboli*[ti] AND (biomarker*[ti] OR marker*[ti] OR predict*[ti] OR indicat*[ti])AND ("1900/01/01"[DP] : "2019/12/31"[DP]) | 7 | 5 |
| 7 | gallbladder | gallbladder[ti] AND (cancer*[ti] OR tumor*[ti] OR carcinoma*[ti]) AND metaboli*[ti] AND (biomarker*[ti] OR marker*[ti] OR predict*[ti] OR indicat*[ti])AND ("1900/01/01"[DP] : "2019/12/31"[DP]) | 0 | 0 |
| 8 | kidney,renal | (kidney[ti] OR renal[ti])AND(cancer*[ti] OR tumor*[ti] OR carcinoma*[ti]) AND metaboli*[ti] AND (biomarker*[ti] OR marker*[ti] OR predict*[ti] OR indicat*[ti]) AND("1900/01/01"[DP] : "2019/12/31"[DP]) | 11 | 3 |
| 9 | larynx | (larynx[ti] OR laryngeal[ti])AND (cancer*[ti] OR tumor*[ti] OR carcinoma*[ti]) AND metaboli*[ti] AND (biomarker*[ti] OR marker*[ti] OR predict*[ti] OR indicat*[ti])AND ("1900/01/01"[DP] : "2019/12/31"[DP]) | 4 | 3 |
| 10 | leukemia | leukemia[ti] AND metaboli*[ti] AND (biomarker*[ti] OR marker*[ti] OR predict*[ti] OR indicat*[ti])AND ("1900/01/01"[DP] : "2018/06/30"[DP]) | 11 | 5 |
| 11 | lip | (lip[ti] OR tongue[ti] OR gum[ti] OR palate[ti] OR parotid[ti] OR mouth[ti])AND (cancer*[ti] OR tumor*[ti] OR carcinoma*[ti]) AND metaboli*[ti] AND (biomarker*[ti] OR marker*[ti] OR predict*[ti] OR indicat*[ti])AND ("1900/01/01"[DP] : "2018/06/30"[DP]) | 1 | 0 |
| 12 | liver | (liver[ti] OR hepatocellular[ti]) AND (cancer*[ti] OR tumor*[ti] OR carcinoma*[ti]) AND metaboli*[ti] AND (biomarker*[ti] OR marker*[ti] OR predict*[ti] OR indicat*[ti])AND ("1900/01/01"[DP] : "2019/12/31"[DP]) | 40 | 18 |
| 13 | lung | lung[ti] AND (cancer*[ti] OR tumor*[ti] OR carcinoma*[ti]) AND metaboli*[ti] AND (biomarker*[ti] OR marker*[ti] OR predict*[ti] OR indicat*[ti])AND ("1900/01/01"[DP] : "2019/12/31"[DP]) | 68 | 36 |
| 14 | lymph | lymphoma[ti]AND (cancer*[ti] OR tumor*[ti] OR carcinoma*[ti]) AND metaboli*[ti] AND (biomarker*[ti] OR marker*[ti] OR predict*[ti] OR indicat*[ti])AND ("1900/01/01"[DP] : "2019/12/31"[DP]) | 13 | 8 |
| 15 | melanoma | melanoma[ti] AND metaboli*[ti] AND (biomarker*[ti] OR marker*[ti] OR predict*[ti] OR indicat*[ti])AND ("1900/01/01"[DP] : "2019/12/31"[DP]) | 14 | 5 |
| 16 | myeloma | myeloma[ti]AND metaboli*[ti] AND (biomarker*[ti] OR marker*[ti] OR predict*[ti] OR indicat*[ti])AND ("1900/01/01"[DP] : "2019/12/31"[DP]) | 14 | 5 |
| 17 | nasopharynx | nasopharynx[ti] AND (cancer*[ti] OR tumor*[ti] OR carcinoma*[ti]) AND metaboli*[ti] AND (biomarker*[ti] OR marker*[ti] OR predict*[ti] OR indicat*[ti])AND ("1900/01/01"[DP] : "2019/12/31"[DP]) | 0 | 0 |
| 18 | oesophagus | oesophagus[ti]AND (cancer*[ti] OR tumor*[ti] OR carcinoma*[ti]) AND metaboli*[ti] AND (biomarker*[ti] OR marker*[ti] OR predict*[ti] OR indicat*[ti])AND ("1900/01/01"[DP] : "2018/06/30"[DP]) | 0 | 0 |
| 19 | ovary | (ovarian[ti] OR ovary[ti]) AND (cancer*[ti] OR tumor*[ti] OR carcinoma*[ti]) AND metaboli*[ti] AND (biomarker*[ti] OR marker*[ti] OR predict*[ti] OR indicat*[ti])AND ("1900/01/01"[DP] : "2019/12/31"[DP]) | 23 | 14 |
| 20 | pancreas | (pancreatic[ti] OR pancreas[ti]) AND (cancer*[ti] OR tumor*[ti] OR carcinoma*[ti]) AND metaboli*[ti] AND (biomarker*[ti] OR marker*[ti] OR predict*[ti] OR indicat*[ti])AND ("1900/01/01"[DP] : "2019/12/31"[DP]) | 20 | 11 |
| 21 | prostate | prostate[ti] AND (cancer*[ti] OR tumor*[ti] OR carcinoma*[ti]) AND metaboli*[ti] AND (biomarker*[ti] OR marker*[ti] OR predict*[ti] OR indicat*[ti]) AND ("1900/01/01"[DP] : "2019/12/31"[DP]) | 47 | 27 |
| 22 | stomach | (stomach[ti] OR gastric[ti]) AND (cancer*[ti] OR tumor*[ti] OR carcinoma*[ti]) AND metaboli*[ti] AND (biomarker*[ti] OR marker*[ti] OR predict*[ti] OR indicat*[ti])AND ("1900/01/01"[DP] : "2019/12/31"[DP]) | 16 | 10 |
| 23 | testis | testis[ti] AND (cancer*[ti] OR tumor*[ti] OR carcinoma*[ti]) AND metaboli*[ti] AND (biomarker*[ti] OR marker*[ti] OR predict*[ti] OR indicat*[ti])AND ("1900/01/01"[DP] : "2018/06/30"[DP]) | 0 | 0 |
| 24 | thyroid | thyroid[ti] AND (cancer*[ti] OR tumor*[ti] OR carcinoma*[ti]) AND metaboli*[ti] AND (biomarker*[ti] OR marker*[ti] OR predict*[ti] OR indicat*[ti])AND ("1900/01/01"[DP] : "2018/06/30"[DP]) | 6 | 2 |
|  |  |  | 503 | 250 |

**Note: Two articles reported two types of cancer**
