# Supplementary material for: CMBD: a manually curated cancer metabolic biomarker knowledge database
Source: Database (Oxford). 2021 Mar 9;2021:baaa094. doi: 10.1093/database/baaa094 (PMC7947571; doi:10.1093/database/baaa094)
Supplement: baaa094_Supp [file baaa094_supp.zip › Supplemental file 2.doc]

**TableS2: HMDB ID of metabolite biomarkers for breast cancer and prostate cancer**

| Breast HMDB ID | Prostate HMDB ID |
| --- | --- |
| HMDB0000063 | HMDB0000094 |
| HMDB0000068 | HMDB0000123 |
| HMDB0000086 | HMDB0000161 |
| HMDB0000122 | HMDB0000162 |
| HMDB0000123 | HMDB0000190 |
| HMDB0000157 | HMDB0000211 |
| HMDB0000158 | HMDB0000252 |
| HMDB0000159 | HMDB0000254 |
| HMDB0000190 | HMDB0000271 |
| HMDB0000210 | HMDB0000510 |
| HMDB0000251 | HMDB0000641 |
| HMDB0000267 | HMDB0000687 |
| HMDB0000569 | HMDB0000851 |
| HMDB0000671 | HMDB0000883 |
| HMDB0000715 | HMDB0000918 |
| HMDB0000791 | HMDB0001256 |
| HMDB0000912 | HMDB0001414 |
| HMDB0000929 | HMDB0001565 |
| HMDB0001419 | HMDB0033923 |
| HMDB0001496 | HMDB0060475 |
| HMDB0001539 |  |
| HMDB0001565 |  |
| HMDB0001961 |  |
| HMDB0002103 |  |
| HMDB0002183 |  |
| HMDB0003269 |  |
| HMDB0006247 |  |
| HMDB0013643 |  |
| HMDB0060475 |  |
| HMDB0060530 |  |
| HMDB0060666 |  |
